# *Monadelpha* (Euphorbiaceae, Plukenetieae), a new genus of Tragiinae from the Amazon rainforest of Venezuela and Brazil

**DOI:** 10.3897/phytokeys.169.59244

**Published:** 2020-12-08

**Authors:** Lynn J. Gillespie, Warren M. Cardinal-McTeague, Kenneth J. Wurdack

**Affiliations:** 1 Research & Collections, Canadian Museum of Nature, P.O. Box 3443, Station D, Ottawa, Ontario, K1P 6P4, Canada Canadian Museum of Nature Ottawa Canada; 2 Agriculture and Agri-Food Canada, Ottawa Research and Development Centre, 960 Carling Ave, Ottawa, Ontario, K1A 0C6, Canada Agriculture and Agri-Food Canada Ottawa Canada; 3 Department of Botany, National Museum of Natural History, Smithsonian Institution, MRC-166, P.O. Box 37012, Washington DC 20013-7012, USA National Museum of Natural History, Smithsonian Institution Washington D.C. United States of America

**Keywords:** Brazil, ITS, molecular phylogeny, Plukenetieae, pollen, *Tragia*, Venezuela

## Abstract

*Monadelpha* L.J.Gillespie & Card.-McTeag., **gen. nov.**, is described as a new member of Euphorbiaceae tribe Plukenetieae subtribe Tragiinae, to accommodate *Tragiaguayanensis*, a species known from western Amazonas, Venezuela and, newly reported here, from Amazonas, Brazil. The genus is unique in the subtribe for having 5-colpate pollen and staminate flowers with filaments entirely connate into an elongate, cylindrical staminal column terminated by a tight cluster of anthers. Phylogenetic analyses based on nuclear rDNA ITS and sampling 156 accessions across the diversity of Tragiinae (all 12 genera and 77 of ~195 species) also support *Monadelpha* as a distinct lineage that is separate from *Tragia*. A revised key to the genera of Tragiinae in South America and Central America is provided.

## Introduction

Members of tribe Plukenetieae are morphologically unusual within Euphorbiaceae for frequently possessing stinging hairs, twining vine or liana habit, and colorful pseudanthia (in *Dalechampia*). The tribe is characterized by apetalous flowers, valvate staminate sepals, and undivided styles that are basally to entirely connate. Plukenetieae contains three subtribes (i.e., Dalechampiinae, Plukenetiinae, Tragiinae), of which Tragiinae is the largest and most diverse, with 12 genera and ~195 species as currently circumscribed (Webster 2014; with updates by Medeiros et al. 2013; Cardinal-McTeague and Gillespie 2016) (Table 1). Subtribe Tragiinae is distinguished from Plukenetiinae by stinging hairs and consistently 3-locular ovaries and eglandular leaves, and from Dalechampiinae by racemose or thyrsoid inflorescences rather than bibracteate pseudanthia (Gillespie 1994a; Webster 2014).

Among Tragiinae, the most species-rich genus is *Tragia*, which includes ~150 species, whereas the other 11 genera are much smaller with only one to 11 species each (Table 1). The complex infrageneric classification of *Tragia* currently comprises two subgenera, six sections (including sect. Monadelphae L.J.Gillespie), one species group, and two unplaced species (Table 1). A single species has been segregated as T.subg.Mauroya (Leandri 1971); however, our preliminary research suggests this species is closely allied with sect. Agirta and does not warrant subgeneric status. All other species belong to T.subg.Tragia. Three other sections, sects. *Leptorhachis* (Klotzsch) Müll.Arg., *Leucandra* (Klotzsch) Müll.Arg. and *Ratiga* Müll.Arg., that are sometimes considered distinct are included here within sect. Tragia, a position supported by pollen (Gillespie 1994a) and molecular studies (Cardinal-McTeague and Gillespie 2016). *Tragia* species exhibit very diverse pollen and floral morphology that is correlated in part with its infrageneric classification (Gillespie 1994a, b). The genus was suggested to be highly paraphyletic based on this morphological diversity (Gillespie 1994a), which is confirmed by recent molecular phylogenetic studies focused on Plukenetieae (Cardinal-McTeague and Gillespie 2016). Three sections of *Tragia*, sects. *Bia* (Klotzch) Müll.Arg., *Ctenomeria* (Harv.) Benth., and *Zuckertia* (Baill.) Müll.Arg., were recently reinstated as genera (Webster 2007, 2014; Medeiros et al. 2013) based on inferences from pollen morphology (Gillespie 1994a), floral morphology, and preliminary molecular results (Wurdack et al. 2005), and are supported by our more in-depth molecular study (Cardinal-McTeague and Gillespie 2016).

**Figure 1.**
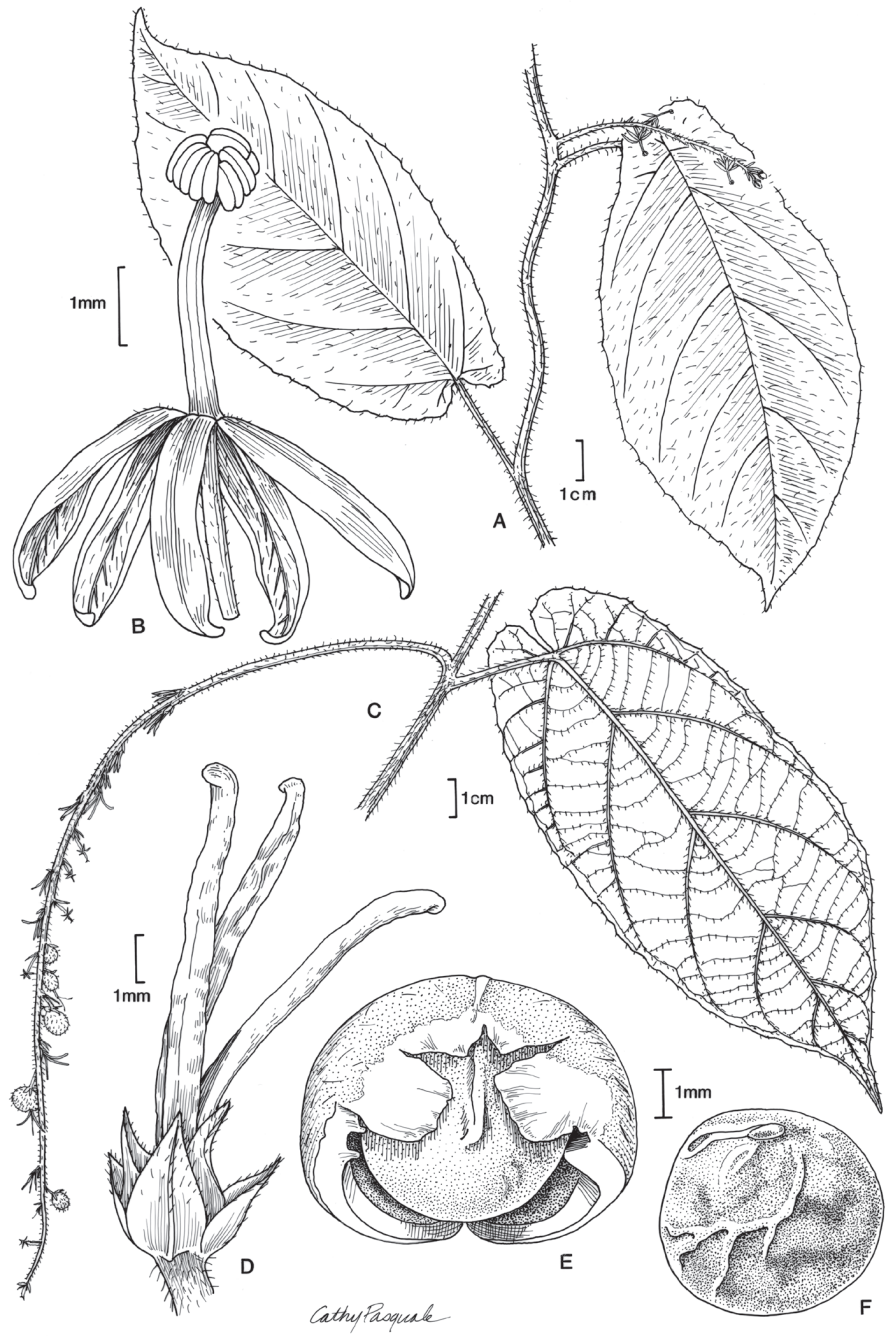
Illustration of *Monadelphaguayanensis*. **A** habit showing staminate inflorescence **B** staminate flower **C** habit showing infructescence **D** pistillate flower **E** mericarp of dehisced capsule with enclosed seed **F** seed, lateral view with hilum at top. Sources: **A, B** based on *Stergios & Aymard 9182* (MO) **C–F** based on *Stergios & Aymard 9182* (NY). Illustration by Cathy Pasquale reproduced from Gillespie (1994b) with permission from the Missouri Botanical Garden Press.

**Figure 2.**
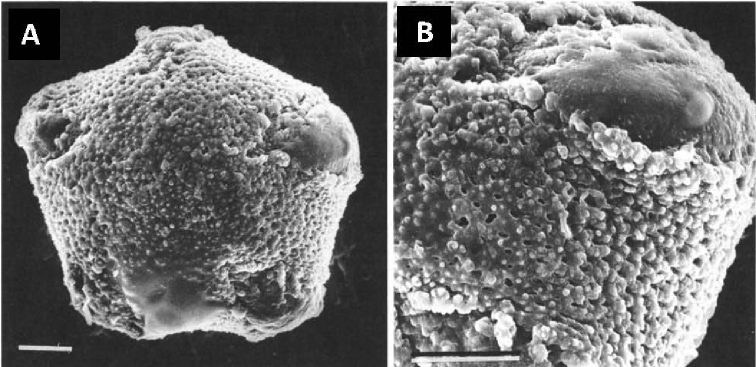
Pollen morphology of *Monadelphaguayanensis***A** SEM of pollen grain showing polar view with five colpi **B** closeup of mesocolpium and two colpi. Source: *Stergios & Aymard 9182* (MO). Figure reproduced from Gillespie (1994b) with permission from the Missouri Botanical Garden Press.

One of the most unusual species of *Tragia* is *T.guayanensis* L.J.Gillespie, which was considered so distinct as to merit its own monotypic section, *Monadelphae* L.J.Gillespie (Gillespie 1994b). The species is characterized by two features unique in Tragiinae: 5-colpate pollen and filaments entirely connate into an elongate cylindrical staminal column (Figs 1, 2). All other *Tragia* species have 3-aperturate pollen, which is mostly 3-colpate, sometimes 3-porate or with three poorly defined apertures (Table 1), with the exception of 4-colpate in *T.rubiginosa* Huft (preliminary observations in Gillespie 1994b), a species unplaced in the sectional classification. Filaments in staminate flowers of *Tragia* are usually distinct to sometimes basally connate. The only other species having filaments entirely connate is *T.lassia* Radcl.-Sm. & Govaerts of sect. Lassia, which has stamens connate into a very short disc-like structure (Baillon 1858: pl. 4, figs 24, 25; pers. obs.), and very different from that of *T.guayanensis*. When describing *T.guayanensis*, Gillespie (1994b) suggested the species was distinct and not closely related to any other *Tragia* species. Nevertheless, she maintained the species within Tragia in its own section pending further study and anticipating that a major reclassification along phylogenetic lines would be necessary.

**Table 1.** Tragiinae genera and infrageneric taxa of *Tragia*: species number, geographic distribution, and pollen morphology. Adapted from Cardinal-McTeague and Gillespie (2016) with pollen characters from Gillespie (1994a, 1994b) and taxonomic updates from this paper.

Genus/section	spp. #	Geographic distribution	Pollen apertures	Pollen tectum
*Acidoton* Sw.	5	Hispaniola, Jamaica	inaperturate	rugulate
*Bia* Klotzsch	5	Costa Rica to South America	inaperturate	foveolate-fossulate or finely reticulate
*Cnesmone* Blume	11	SE Asia	weakly 3-colpate	punctate
*Ctenomeria* Harv.	2	South Africa	weakly 3-aperturate	finely foveolate-reticulate
*Gitara* Pax & K.Hoffm.	1	Central and South America	3-colpate	finely foveolate-reticulate
*Megistostigma* Hook.f.	5	SE Asia	weakly 3-colpate to inaperturate	punctate
*Monadelpha* L.J.Gillespie & Card.-McTeag., gen. nov.	1	Venezuela (Amazonas), Brazil (Amazonas)	5-colpate	foveolate
*Pachystylidium* Pax & K.Hoffm.	1	SE Asia	weakly 3-porate	punctate
*Platygyna* P.Mercier	7	Cuba	inaperturate	reticulate or rugulate
*Sphaerostylis* Baill.	2	Madagascar	unknown	unknown
*Tragia* L.	~150	Pantropical to warm temperate		
sect. Agirta Baill.	5	Madagascar	unknown	unknown
sect. Lassia (Baill.) Müll.Arg.	2	Madagascar	3-colpate	reticulate
sect. Leptobotrys (Baill.) Müll.Arg.	2	SE USA	weakly 3-porate	punctate
sect. Tagira Müll.Arg.	82	Africa, Madagascar, S Asia	3-colpate	reticulate
sect. Tragia	53	S USA to South America, Caribbean	3-colpate	intectate-baculate
Australian species group	3	Australia	3-porate	punctate
*T.biflora* Urb. & Ekman (unplaced)	1	Hispaniola	unknown	unknown
*T.rubiginosa* Huft (unplaced)	1	Venezuela	4-colpate	punctate
subg. Mauroya Leandri	1	Madagascar	weakly 3-aperturate	finely reticulate
*Tragiella* Pax & K.Hoffm.	4	E and S Africa	3-colpate	reticulate
*Zuckertia* Baill.	2	Mexico, Central America	3-colpate	finely reticulate

Here we present molecular phylogenetic results placing *T.guayanensis* within subtribe Tragiinae that supports its recognition as a distinct genus. The new genus *Monadelpha* is described for *T.guayanensis* based on its unique pollen and floral morphology and isolated phylogenetic position within Tragiinae. This is the first of several contributions towards a new phylogenetic classification of subtribe Tragiinae.

## Material and methods

### Molecular phylogeny

To determine the phylogenetic relationships of *Monadelpha*, we sequenced and analyzed the Internal Transcribed Spacer (ITS) region (including complete ITS1, 5.8S, and ITS2, and flanking portions of 18S and 26S) of nuclear rDNA. ITS has been shown to provide good resolution of Tragiinae in the prior studies (e.g., Cardinal-McTeague and Gillespie 2016; Cardinal-McTeague et al. 2019) from which our core taxon sampling is drawn and presents few alignment problems across genera. Orthologous plastid data could not be recovered from the degraded *Monadelpha* sample, but the limited phylogenetic resolution of the more slowly evolving plastid loci is established and their addition would be unlikely to change our findings. Our taxon sampling of 156 accessions included 77 of ~195 species of Tragiinae (39% of total diversity) with representatives of all 12 Tragiinae genera and seven of the eight sections/species groups in *Tragia* (excluding the Madagascan subg. Mauroya, only known from its type collection of *T.ivohibeensis* Leandri). Due to unusually high GC content in the close relatives of Tragiinae (which results in challenging DNA alignments and recovers some questionable relationships; Cardinal-McTeague, unpublished data), we rooted the tree using three accessions from the sister clade of Plukenetieae, which contains tribes Bernardieae and Caryodendreae (Wurdack et al. 2005; Cervantes et al. 2016).

Extractions of genomic DNA, fluorescent Sanger sequencing, and contig assembly for the ITS sequences followed the protocols of previous molecular studies in Plukenetieae (Cardinal-McTeague and Gillespie 2016; Cardinal-McTeague et al. 2019). The paratype of *T.guayanensis* (*Williams 14990*, US) was sampled and sequenced at the Smithsonian separately from all other new data, under more stringent conditions for degraded museum samples following protocols in Dorr et al. (2018). That specimen is well preserved and the data appear authentic based on appropriate negative controls and unique phylogenetic placement. The sequences were aligned using the auto-select algorithm of MAFFT ver. 7.450 (Katoh and Standley 2013) in Geneious ver. 11.1.5 (BioMatters, Auckland, New Zealand), and the optimal model of nucleotide evolution was ranked by AIC (Akaike Information Criterion) using default search parameters across three substitution schemes in jModeltest2 ver. 2.1.6 on XSEDE (Darriba et al. 2012; Miller et al. 2010). Subsequent analyses were conducted on all data in the alignment and potentially ambiguous regions were few.

We estimated a phylogenetic tree using Bayesian inference with MrBayes ver. 3.2.6 on XSEDE (Ronquist et al. 2012), executing an (MC)^3^ analysis with two runs of 3 million generations and sampling every 1000 generations, using the optimal model of nucleotide evolution on an unpartitioned alignment (remaining parameters as default). Runs were considered converged if ESS (effective sample size) of each parameter were >500 in Tracer ver. 1.7 (Rambaut et al. 2018), and if PSRF (potential scale reduction factor) and the standard deviation of split frequencies were close to 1.0 and <0.005, respectively, as determined by the MrBayes output. A 50% majority rule consensus tree was calculated following a 25% burn-in, resulting in Bayesian posterior probability (PP) values based on posterior distribution of 4500 trees from the combined runs. For an additional estimate of branch support, we inferred maximum likelihood bootstrap percentages (MLBP) using 1000 rapid bootstrap replicates under default parameters with RAxML-HPC ver. 8 on XSEDE (Stamatakis 2014). In the Results, we interpret strong branch support as PP >0.95 and MLBP >85. Discussion of the subclades (T1–T10) follows the naming convention of Cardinal-McTeague and Gillespie (2016) with minor adjustments.

### Data resources

The data underpinning the analyses reported in this paper (DNA alignment and resulting Bayesian tree) are deposited in the Dryad Data Repository at https://doi.org/10.5061/dryad.5hqbzkh4d.

### Morphology

Specimens were examined at CAN and US, on loan from MO, NY, and P (herbarium acronyms following Index Herbariorum (http://sweetgum.nybg.org/science/ih/), and from other herbaria via online images in the Global Biodiversity Information Facility (GBIF.org 01 Oct 2020 Occurrence Download https://doi.org/10.15468/dl.upmgky). The key was adapted from Gillespie (1994b), modified and updated based on examination of specimens and the following references: Mulgura de Romero and Gutierrez de Sanguinetti (1989); Radcliffe-Smith (2001); Medeiros et al. (2013); Steinmann and Ramírez-Amezcua (2013); Cardinal-McTeague and Gillespie (2016); Webster (2014).

## Phylogenetic results

**Figure 3.**
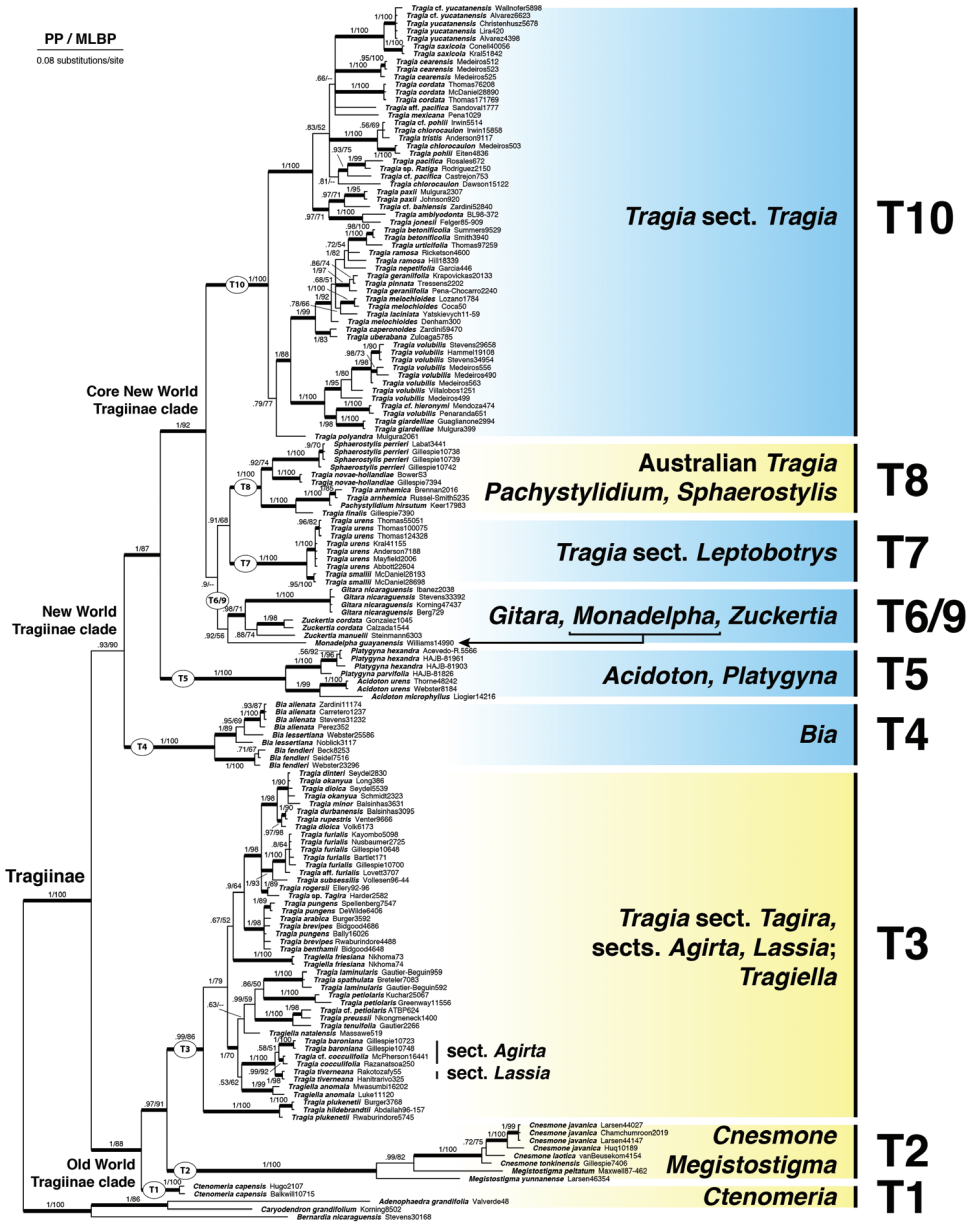
Phylogenetic relationships of *Monadelpha*. Bayesian 50% majority rule consensus tree for the 159-accession (80 taxa) ITS dataset of Tragiinae, demonstrating the distinct phylogenetic placement of *Monadelpha* (subclade T6/9). Subclade naming system follows Cardinal-McTeague and Gillespie (2016) with minor adjustments. Branches are labeled with Bayesian posterior probabilities (PP) and maximum likelihood bootstrap percentages (MLBP). Bold branches indicate strong support (PP >0.95, MLBP >85) and coloured boxes indicate general distribution (New World = blue, Old World = yellow).

Our 159-terminal (80 taxa) ITS dataset, including 55 new sequences, had an aligned length of 795 characters (410 variable, 353 parsimony informative [44%], 0.7% missing data), and GTR+I+G was identified as its optimal model of nucleotide evolution. Bayesian and ML analyses revealed very similar results. The 50% majority rule Bayesian topology was well resolved with most clades strongly supported by PP and MLBP (Fig. 3).

The phylogeny is divided into two major clades with strong to moderate support, the Old World Tragiinae clade (T1–T3) and the New World Tragiinae clade (T4–T10). The resolution of subclades T1–T3 was strongly supported, with subclade T1 (*Ctenomeria*) sister to T2 (*Cnesmone*, *Megistostigma*) + T3 (Tragiasect.Tagira, embedded with *T.* sects. *Agirta* and *Lassia*, and *Tragiella*). Subclades T4–T10 were mostly strongly supported, with the exception of the modified subclade T6/9, which includes the new genus *Monadelpha* with moderate support (PP = 0.92, MLBP = 56). The New World Tragiinae clade contains a small successive grade of subclades T4 (*Bia*) and T5 (*Acidoton*, *Platygyna*) that culminates into the strongly supported Core New World Tragiinae clade (T6–T10). This core clade comprises a weakly supported clade (PP = 0.90, MLBP <50) with three distinctive subclades, T6/9 (*Gitara*, *Monadelpha*, and *Zuckertia*) sister to T7 (Tragiasect.Leptobotrys) + T8 (Australian *Tragia*, *Pachystylidium*, and *Sphaerostylis*), which together are sister to the large subclade T10 (Tragiasect.Tragia). *Monadelpha* is on a long branch, moderately supported as sister to *Zuckertia* + *Gitara* (PP = 0.92, MLBP = 56), and well separated from *Tragia* and other Tragiinae genera.

## Discussion

The phylogenetic relationships of Tragiinae recovered here largely agree with previous phylogenetic analyses of Plukenetieae based on ITS and plastid *psbA-trnH* data (Cardinal-McTeague and Gillespie 2016). Our increased Tragiinae taxon sampling (77 here compared to 50 previously) improved both resolution and support, despite including only ITS data. A noteworthy difference is the revised placement of *Gitara* (subclade T9, formerly weakly supported as sister to Tragiasect.Tragia, T10; Cardinal-McTeague and Gillespie 2016), which is here strongly supported as sister to *Zuckertia* (T6), with *Monadelpha* sister to both of them. *Monadelpha* is an isolated lineage, clearly distinct from New World and Old World clades of *Tragia*. Support for its relationship with *Gitara* and *Zuckertia* (subclade T6/9) is not strong, which suggests that its position on the phylogeny may not be stable and could vary with additional sequence data. Inclusion of the *Monadelpha*ITS sequence in a broader analysis of Plukenetieae that sampled six loci (nuclear ribosomal ETS, ITS; low copy *KEA1*, *TEB*; plastid *matK*, *ndhF*; results not shown) recovered similar results with strong support for subclade T6/9, the inclusion of *Monadelpha* in subclade T6/9, and with weak support for generic relationships therein (Cardinal-McTeague et al., unpublished).

The 5-colpate pollen of *Monadelpha* is unique among Plukenetieae. All other Plukenetieae have 3-aperturate or inaperturate pollen, with the exception of *T.rubiginosa* (discussed below). Among New World Tragiinae, *Gitara*, *Zuckertia*, and Tragiasect.Tragia pollen is 3-colpate, whereas pollen of *Acidoton*, *Bia*, and *Platygyna* is inaperturate, and T.sect.Leptobotrys is 3-porate (Gillespie 1994a, 1994b) (Table 1). *Monadelpha* shares uneven colpus margins with the 3-colpate taxa. Its tectate-foveolate exine is more similar to the tectate and finely fossulate-reticulate or finely reticulate exines of *Gitara* and *Zuckertia* than to the intectate-baculate exine of Tragiasect.Tragia.

*Monadelpha* is also morphologically distinct, especially its staminate flowers with filaments completely connate into a long cylindrical staminal column bearing a tight terminal cluster of ±5 anthers. *Acidoton*, *Bia*, *Gitara*, *Platygyna*, and *Zuckertia* all have numerous free stamens, the large *Tragia* sects. *Tragia* and *Tagira* have 3 stamens (sometimes more, to 22) that are distinct or connate only at the base, and T.sect.Leptobotrys has two stamens (rarely 3) connate basally. Only the distantly related *Tragialassia* (T.sect.Lassia) of Madagascar has stamens or filaments entirely connate, but this feature has obviously evolved independently. Its androecium, consisting of a small 3-anthered disc-shaped structure on a very short narrow column, is very different from that of *Monadelpha*.

Other characters of *Monadelpha* that are unusual for Tragiinae include unisexual inflorescences and long, mostly distinct styles. Whereas most Tragiinae have bisexual inflorescences with pistillate flowers proximal, *Monadelpha* shares unisexual inflorescences with *Gitara* and the Caribbean genera *Acidoton* and *Platygyna* (plus a few species of Old World Tragiinae). Styles of *Monadelpha* are slender, cylindrical, mostly smooth (papillose only at the apex), up to 10 mm long, and connate basally (up to ¼ their length). Most New World Tragiinae and Old World *Tragia* have styles that are much shorter, relatively thicker, and basally to mostly connate into a thick stylar column. Perhaps most similar is *Zuckertia* with somewhat longer (to 5 mm), slender cylindrical styles that are connate into a slender column, but differ in the degree of connation (1/2–3/4 their length) and the free portion papillose adaxially.

*Tragiarubiginosa* from Amazonian Peru is another morphologically unusual *Tragia* species (Huft 1989) that is unplaced in the present sectional classification. The species has 4-colpate pollen (preliminary observations in Gillespie 1994b) and is the only species in Tragiinae other than *Monadelpha* with a pollen aperture number greater than three. Its broad, thick, subsessile stigmas are unique in Tragiinae and its staminate flowers with five sessile anthers are unusual. The species is morphologically distinct from *Monadelpha*, and the two taxa are unlikely to be closely related. Molecular data is not yet available to determine its phylogenetic position within Tragiinae.

The isolated phylogenetic position (including a long branch length), and accompanying distinctive stamen and pollen morphology all support the recognition of *Monadelpha* as a new genus separate from *Tragia*. Our results suggest a possible relationship with the northern South American and Central American monotypic genus *Gitara* and the Mexican and Central American ditypic genus *Zuckertia*.

## Taxonomic treatment

### Monadelpha

Taxon classificationPlantaeMalpighialesEuphorbiaceae

L.J.Gillespie & Card.-McTeag. gen. nov.

29001A7D-7D95-55A6-8243-163EA39A7711

urn:lsid:ipni.org:names:77213225-1

Tragia section Monadelphae L.J.Gillespie, Novon 4: 331. 1994.

#### Diagnosis.

Similar to *Tragia* and other Tragiinae genera but differing in having 5-colpate pollen and monadelphous stamens with filaments entirely connate into an elongate, cylindrical staminal column with anthers tightly clustered together at apex.

#### Type and only known species.

*Monadelphaguayanensis* (L.J.Gillespie) L.J.Gillespie & Card.-McTeag.

#### Description.

*Habit* climbing vines, apparently monoecious; latex absent; stems twining; stems, leaves and inflorescences with stinging and simple hairs. *Stipules* narrowly triangular or lanceolate, small, caducous. *Leaves* simple, alternate, evergreen, petiolate, eglandular; blades elliptic, ovate-elliptic, broadly elliptic, broadly ovate-elliptic, or suborbicular, chartaceous, apex acuminate, base narrowly cordate, margins irregularly serrulate or denticulate with minute glandular setae, venation pinnate; petiolar and laminar glands absent. *Inflorescences* slender racemes, unisexual, flowers single per node in bract axil; bracts small, lanceolate or narrowly lanceolate, eglandular; staminate inflorescence axillary; pistillate inflorescence (known only in fruiting stage) terminal but appearing leaf-opposed. *Staminate flowers* pedicellate; sepals 5, narrowly oblong, valvate; corolla and disc absent; stamens apparently 5, monadelphous; filaments connate into an elongate, ±cylindrical staminal column, bearing a dense cluster of ±5 anthers; pollen 5-colpate, oblate-spheroidal to suboblate, amb pentagonal, exine tectate-perforate, tectum foveolate and microverrucate, colpi with uneven margins. *Pistillate flowers* (description based on old flowers on infructescence axis) pedicellate; sepals 6, ovate, distinctly imbricate, margins entire; corolla and disc absent; ovary 3-locular with 1 ovule per locule, 3-lobed, densely covered with stinging hairs; styles 3, long-cylindrical, mostly distinct, connate basally for 10–25% length, papillose at apex. *Fruits* 3-lobed capsules, dehiscing into 3 bivalved mericarps; pericarp woody, sparsely covered with stinging hairs; columella persistent, with 3 perpendicular apical arms; seeds 3, subglobose, abaxial surface somewhat obtusely angular, ecarunculate; sepals persistent.

#### Etymology.

The genus name is combined from *monos* (Greek, one) and *adelphos* (Greek, brother), and refers to monadelphous with filaments united and to TragiasectionMonadelphae.

### Monadelpha guayanensis

Taxon classificationPlantaeMalpighialesEuphorbiaceae

(L.J.Gillespie) L.J.Gillespie & Card.-McTeag. comb. nov.

30314F57-75C4-5DAA-A856-DEE5AFA155EB

urn:lsid:ipni.org:names:77213226-1

Figures 1 , 2

Tragia guayanensis L.J.Gillespie, Novon 4: 330–338. 1994.

#### Type.

Venezuela. Amazonas: Río Casiquiare entre la boca del [Rio] Siapa y el caño Momoni, 18 Feb–4 Mar 1986, *B. Stergios & G. Aymard 9182* (holotype: MO-260419! – staminate; isotype: NY-00076710! – pistillate).

#### Description.

See Gillespie (1994b). Emended here (based on *Ule 5013*): *Leaves*: petiole 2–8 cm long, blade elliptic, ovate-elliptic, broadly elliptic, broadly ovate-elliptic, or suborbicular, 12–25 × 6–17 cm, apex with acumen 1–2 cm long, base cordate with narrow sinus 0.8–2.5 cm deep, margins irregularly serrulate or denticulate. *Staminate inflorescences* ~3–18 cm long.

#### Etymology.

The specific epithet is derived from Guayana, and refers both to the Guayana Shield region of northern South America and to the Guayana Region of Venezuela where the species is native. Guayana is of Amerindian derivation by European colonists, and may come from the tribe Guayanos or the Indigenous word *uayana*, meaning pale (see Berry et al. 1995).

#### Additional collections examined.

Brazil. Amazonas: Rio Juruá, Nov 1900, *E.H.G. Ule 5013* (L-0160690, K-001205092). Venezuela. Amazonas: En la isla de Trapichote, Delta del Ventuari, [3°57'31.45"N, 67°12'7.45"W], alt. 125 m, 21 Apr 1942, *L. Williams 14990* (paratypes: F-1189188, US-1833601).

#### Distribution and preliminary conservation status.

Known from only three collections. The two from Venezuela are ~220 km apart in lowland rainforests of the upper Orinoco Basin and Río Casiquiare of western Amazonas. The Brazilian collection occurs at least 500 km to the south along the Rio Juruá (locality imprecise) in Amazonas. They occur in remote, pristine rainforest and their IUCN Red List Category presently should be Data Deficient given limited information of distribution and threats.

#### Notes.

*Monadelphaguayanensis* is newly reported here from Brazil based on one 1900 collection by E.H.G. Ule. Staminate inflorescences on this collection (L-0160690 sheet) are considerably longer than previously described, and are closer in length to the pistillate inflorescence. It appears that the staminate inflorescence on the holotype may be damaged and partly missing or possibly less mature. Leaf blades are more variable in size and shape than on the two Venezuelan collections, some blades being very similar, others larger and relatively broader.

*Monadelphaguayanensis* has unisexual inflorescences and is likely monoecious (rather than dioecious). Although unisexual inflorescences are not found on the same branch, the type collection, *Stergios & Aymard 9182*, has inflorescences of both sexes, and is thus monoecious if one assumes branches originate from a single individual. Further collections are needed to confirm this character.

### Key to Tragiinae in South America and Central America

**Table:** 

1	Filaments absent or entirely connate into an elongate staminal column; pollen 4- or 5-colpate; inflorescences unisexual, racemose; staminate flowers with 5 sepals and 5 stamens	**2**
–	Filaments distinct or rarely partly connate; pollen 3-colpate, weakly 3-porate, or inaperturate; inflorescences bisexual (unisexual in *Gitara*), racemose or paniculate with a single branch; staminate flowers with 3–5 (6) sepals and (1) 2–40+ stamens	**3**
2	Anthers in a dense cluster on an elongate ±cylindrical staminal column; pollen 5-colpate; styles cylindrical, 6–10 mm long	***Monadelpha***
–	Anthers sessile; pollen 4-colpate; stigmas subsessile, broad	***Tragiarubiginosa***
3	Inflorescences unisexual; dioecious, erect shrubs; anther connective with tuft of stinging hairs	***Gitara***
–	Inflorescences bisexual with pistillate flowers basal; monecious vines, herbs, or subshrubs; anther connective lacking tuft of stinging hairs or present but minute	**4**
4	Inflorescences racemose, with 1 (2) pistillate flower(s) at the basal 1 (–9) node(s) (*T.polyandra* with (1) 2–4 (5) flowers on a short basal branch); stamens (1) 2–5 (–22); staminate disc usually absent, if present comprising a single central structure; pollen exine intectate, baculate	**Tragia (sect. Tragia)**
–	Inflorescences consisting of a racemose staminate main axis and a single elongate basal branch bearing 5–30 pistillate flowers (branch short with (1) 2–4 flowers in *Zuckertiamanuelii*); stamens 6–40+; staminate disc segmented or absent; pollen exine tectate, finely reticulate or foveolate-fossulate	**5**
5	Staminate flowers with 3 (4) sepals, 5–10 disc segments, and 6–20 stamens; leaf blades 5–16 cm long, usually unlobed; pollen inaperturate; South America to Costa Rica	***Bia***
–	Staminate flowers with 5 or 6 sepals, no disc, and 17–40+ stamens; leaf blades (7–) 12–25 cm long, unlobed to 3-lobed; pollen tricolpate; Mexico and Central America	***Zuckertia***

## Supplementary Material

XML Treatment for Monadelpha

XML Treatment for Monadelpha guayanensis

## Appendix 1

Sources for ITS data used in phylogenetic analyses. Voucher data (country, collector, number, herbarium code) are provided, with new GenBank numbers in bold and beginning with MK and MW.

**Outgroup**: *Adenophaedragrandifolia* (Klotzsch) Müll.Arg. Costa Rica • Valverde 48 (NY), **MK780909**. *Bernardianicaraguensis* Standl. & L.O.Williams Nicaragua • Stevens 30168 (MO), KP794419. *Caryodendrongrandifolium* (Müll.Arg.) Pax Ecuador • Korning 8502 (MO), KP794421. **Ingroup**: *Acidotonmicrophyllus* Urban Dominican Republic • Liogier 14216 (NY), **MK780907**. *Acidotonurens* Sw. Jamaica • Thorne 48242 (MO), KP794316. *Acidotonurens* Sw. Jamaica • Webster 8184 (DAV), **MK780908**. *Biaalienata* Didr. Bolivia • Carretero 1237 (MO), KP794320. *Biaalienata* Didr. Paraguay • Pérez 352 (MO), KP794318. *Biaalienata* Didr. Paraguay • Stevens et al. 31232 (MO), **MK780928**. *Biaalienata* Didr. Paraguay • Zardini 11174 (MO), KP794319. *Biafendleri* Müll.Arg. Bolivia • Beck 8253 (MO), KP794321. *Biafendleri* Müll.Arg. Paraguay • Seidel and Herzog 7516 (DAV), **MK780930**. *Biafendleri* Müll.Arg. Peru • Webster 23296 (DAV), **MK780929**. Biacf.lessertiana Brazil • Noblick 3117 (DAV), **MK780931**. *Bialessertiana* Baill. Brazil • Webster 25586 (DAV), **MK780932**. *Cnesmonejavanica* Blume Bangladesh • Huq 10189 (US), **MK780937**. *Cnesmonejavanica* Blume Thailand • Chamchumroon 2019 (L), KP794430. *Cnesmonejavanica* Blume Thailand • Larsen 44027 (MO), KP794427. *Cnesmonejavanica* Blume Thailand • Larsen 44147 (MO), KP794428. *Cnesmonelaotica* (Gagnep.) Croizat Thailand • van Beusekom 4154 (L), KP794431. *Cnesmonetonkinensis* (Gagnep.) Croizat Vietnam • Gillespie 7406 (CAN), **MK780938**. *Ctenomeriacapensis* (Thunb.) Harv. ex Sond. South Africa • Balkwill and Balkwill 10715 (MO), **MK780939**. *Ctenomeriacapensis* (Thund.) Harv. ex Sond. South Africa • Hugo 2107 (MO), KP794322. *Gitaranicaraguensis* (Hemsl.) Card.-McTeag. & L.J.Gillespie Brazil • Berg et al. 729 (CAN), **MK780953**. *Gitaranicaraguensis* (Hemsl.) Card.-McTeag. & L.J.Gillespie Ecuador • Korning 47437 (MO), KP794398. *Gitaranicaraguensis* (Hemsl.) Card.-McTeag. & L.J.Gillespie Nicaragua • Stevens 33392 (MO), **MK780954**. *Gitaranicaraguensis* (Hemsl.) Card.-McTeag. & L.J.Gillespie Panama • Ibañez 2038 (MO), KP794397. *Megistostigmapeltatum* (J.J.Sm.) Croizat Thailand • Maxwell 87-462 (L), **MK780958**. *Megistostigmayunnanense* Croizat Thailand • Larsen 46354 (L), **MK780959**. *Monadelphaguayanensis* (L.J.Gillespie) L.J.Gillespie & Card.-McTeag. Venezuela • Williams 14990 (US-1833601), **MW264490**. *Pachystylidiumhirsutum* (Blume) Pax & K.Hoffm. Thailand • Keer 17893 (L), KP794408. *Platygynahexandra* (Jacq.) Müll.Arg. Cuba • Acevedo-Rodríguez 5566 (NY), KP794317. *Platygynahexandra* (Jacq.) Müll.Arg. Cuba • HAJB 81903 (MICH), **MK780963**. *Platygynahexandra* (Jacq.) Müll.Arg. Cuba • HAJB 81961 (MICH), **MK780964**. *Platygynaparvifolia* Alain Cuba • HAJB 81826 (MICH), **MK780965MK780965**. *Sphaerostylisperrieri* Leandri Madagascar • Gillespie 10738 (CAN), KP794413. *Sphaerostylisperrieri* Leandri Madagascar • Gillespie 10739 (CAN), KP794414. *Sphaerostylisperrieri* Leandri Madagascar • Gillespie 10742 (CAN), KP794415. *Sphaerostylisperrieri* Leandri Madagascar • Labat 3441 (MO), KP794412. Tragiaaff.furialis Tanzania • Lovett 3707 (MO), KP794344. Tragiaaff.pacifica EL Salvador • Sandoval 1777 (MO), KP794388. *Tragiaamblyodonta* (Müll.Arg.) Pax & K.Hoffm. USA • B.L. 98-372 (MO), KP794376. *Tragiaarabica* (Müll.Arg.) Baill. ex Prain Ethiopia • Burger 3592 (US), **MK780910**. *Tragiaarnhemica* P.I.Forst. Australia • Brennan 2016 (DNA), KP794410. *Tragiaarnhemica* P.I.Forst. Australia • Russel-Smith 5235 (DNA), KP794409. *Tragiabaroniana* Prain Madagascar • Gillespie et al. 10723 (CAN), **MK780911**. *Tragiabaroniana* Prain Madagascar • Gillespie et al. 10748 (CAN), **MK780912**. *Tragiabenthamii* Baker Tanzania • Bidgood 4648 (MO), KP794328. *Tragiabetonicifolia* Nutt. USA • Smith 3940 (MO), KP794364. *Tragiabetonicifolia* Nutt. USA • Summers 9529 (MO), KP794363. *Tragiabrevipes* Pax Tanzania • Bidgood 4686 (MO), KP794325. *Tragiabrevipes* Pax Uganda • Rwaburindore 4488 (MO), KP794329. *Tragiacaperonoides* Pax & K.Hoffm. Paraguay • Zardini and Gamarra 59470 (MO), **MK780967**. *Tragiacearensis* Pax & K.Hoffm. Brazil • Medeiros et al. 512 (R), **MK780968**. *Tragiacearensis* Pax & K.Hoffm. Brazil • Medeiros et al. 523 (R), **MK780969**. *Tragiacearensis* Pax & K.Hoffm. Brazil • Medeiros et al. 525 (R), **MK780970**. Tragiacf.bahiensis Paraguay • Zardini 52840 (MO), KP794378. Tragiacf.cocculifolia Madagascar • McPherson and van der Werff 16441 (CAN), **MK780913**. Tragiacf.hieronymi Bolivia • Mendoza 474 (NY), **MK780975**. Tragiacf.pacifica Mexico • Castrejón 753 (MO), KP794392. Tragiacf.petiolaris Uganda • ATBP 624 (MO), KP794351. Tragiacf.pohlii Brazil • Irwin 5514 (NY), **MK780971**. Tragiacf.yucatanensis Mexico • Álvarez 6623 (MO), KP794383. Tragiacf.yucatanensis Guatemala • Wallnöfer 5898 (MO), KP794381. *Tragiachlorocaulon* Baill. Brazil • Dawson 15122 (MO), KP794390. *Tragiachlorocaulon* Baill. Brazil • Irwin 15858 (MO), KP794391. *Tragiachlorocaulon* Baill. Brazil • Medeiros et al. 503 (R), **MK780973**. *Tragiacocculifolia* Prain Madagascar • Razanatsoa 250 (MO), KP794331. *Tragiacordata* Michx. USA • McDaniel 28890 (MO), KP794395. *Tragiacordata* Michx. USA • Thomas 171769 (MO), KP794396. *Tragiacordata* Michx. USA • Thomas 76208 (CAN), KP794394. *Tragiadinteri* Pax Namibia • Seydel 2830 (L), **MK780914**. *Tragiadioica* Sond. Namibia • Seydel 5539 (MO), KP794335. *Tragiadioica* Sond. Namibia • Volk 6173 (MO), KP794332. *Tragiadurbanensis* Kuntze South Africa • Balsinhas 3095 (MO), KP794333. *Tragiafinalis* P.I.Forst. Australia • Gillespie 7390 (CAN), KP794411. *Tragiafurialis* Bojer ex Prain Madagascar • Gillespie 10648 (CAN), KP794342. *Tragiafurialis* Bojer ex Prain Madagascar • Gillespie 10700 (CAN), **MK780917**. *Tragiafurialis* Bojer ex Prain Madagascar • Nusbaumer 2725 (MO), KP794343. *Tragiafurialis* Bojer ex Prain Mayotte • Barthlet 171 (MO), KP794341. *Tragiafurialis* Bojer ex Prain Tanzania • Kayombo 5098 (MO), KP794340. *Tragiageraniifolia* Klotzsch ex Baill. Argentina • Krapovickas 20133 (MO), KP794368. *Tragiageraniifolia* Klotzsch ex Baill. Paraguay • Peña-Chocarro 2240 (MO), KP794370. *Tragiagiardelliae* M.M.Gutiérrez & M.E.Múlgura Argentina • Guaglianone 2994 (MO), KP794360. *Tragiagiardelliae* M.M.Gutiérrez & M.E.Múlgura Argentina • Mulgura 399 (NY), **MK780974**. *Tragiahildebrandtii* Müll.Arg. Tanzania • Abdallah 96/157 (MO), KP794355. *Tragiajonesii* Radcl.-Sm. & Govaerts Mexico • Felger 85-909 (MO), KP794377. *Tragialaciniata* (Torr.) Müll.Arg. Mexico • Yatskievych 11-59 (MO), **MK780976**. *Tragialaminularis* Müll.Arg. Côte D’Ivoire • Gautier-Béguin 592 (MO), KP794349. *Tragialaminularis* Müll.Arg. Côte D’Ivoire • Gautier-Béguin 959 (MO), KP794348. *Tragiamelochioides* Griseb. Bolivia • Coca 50 (MO), KP794373. *Tragiamelochioides* Griseb. Bolivia • Lozano 1784 (MO), KP794372. *Tragiamelochioides* Griseb. Uruguay • Denham et al. 300 (SI), **MK780978**. *Tragiamexicana* Müll.Arg. Belize • Peña 1029 (MO), KP794393. *Tragiaminor* Sond. South Africa • Balsinhas 3631 (MO), KP794338. *Tragianepetifolia* Cav. Mexico • García 446 (MO), KP794366. *Tragianovae-hollandiae* Müll.Arg. Australia • Bower S3 (CAN), **MK780960**. *Tragianovae-hollandiae* Müll.Arg. Australia • Gillespie 7394 (CAN), KP794417. *Tragiaokanyua* Pax Botswana • Long 386 (MO), KP794336. *Tragiaokanyua* Pax Zambia • Schmidt 2323 (MO), KP794337. *Tragiapacifica* McVaugh EL Salvador • Rosales 672 (MO), KP794389. *Tragiapaxii* Lourteig & O’Donnell Argentina • Johnson 920 (MO), KP794380. *Tragiapaxii* Lourteig & O’Donnell Argentina • Múlgura 2307 (MO), KP794379. *Tragiapetiolaris* Radcl.-Sm. Tanzania • Greenway and Polhill 11556 (L), **MK780920**. *Tragiapetiolaris* Radcl.-Sm. Tanzania • Kuchar 25067 (MO), KP794354. *Tragiapinnata* (Poir.) A.Juss. Argentina • Tressens 2202 (MO), KP794369. *Tragiaplukenetii* Radcl.-Sm. Ethiopia • Burger 3768 (US), **MK780919**. *Tragiaplukenetii* Radcl.-Sm. Uganda • Rwaburindore 5745 (MO), KP794356. *Tragiapohlii* Müll.Arg. Brazil • Eiten 4836 (UBC), **MK780979**. *Tragiapolyandra* Vell. Argentina • Múlgura 2061 (MO), KP794375. *Tragiapreussii* Pax Cameroon • Nkongmeneck 1400 (MO), KP794352. *Tragiapungens* (Forssk.) Müll.Arg. Ethiopia • De Wilde 6406 (MO), KP794327. *Tragiapungens* (Forssk.) Müll.Arg. Somalia • Bally 16026 (MO), KP794326. *Tragiapungens* (Forssk.) Müll.Arg. Yemen • Spellenberg 7547 (L), **MK780921**. *Tragiaramosa* Torr. USA • Hill 18339 (MO), KP794371. *Tragiaramosa* Torr. USA • Ricketson 4600 (MO), KP794367. *Tragiarogersii* Prain South Africa • Ellery 92/96 (MO), KP794339. *Tragiarupestris* Sond. South Africa • Venter 9666 (MO), KP794334. *Tragiasaxicola* Small USA • Conell 40056 (MO), KP794386. *Tragiasaxicola* Small USA • Kral 51842 (MO), KP794387. *Tragiasmallii* Shinners USA • McDaniel 28193 (MO), KP794401. *Tragiasmallii* Shinners USA • McDaniel 28698 (MO), KP794402. *Tragia* sp. *Ratiga* EL Salvador • Rodriguez and Tejada 2150 (MO), **MK780980**. *Tragia* sp. *Tagira* Zambia • Harder and Bingham 2582 (CAN), **MK780923**. *Tragiaspathulata* Benth. Togo • Breteler 7083 (MO), KP794350. *Tragiasubsessilis* Pax Tanzania • Vollesen 96/44 (MO), KP794345. *Tragiatenuifolia* Benth. Côte D’Ivoire • Gautier 2266 (MO), KP794353. *Tragiativerneana* Leandri Madagascar • Hanitrarivo 325 (CAN), **MK780925**. *Tragiativerneana* Leandri Madagascar • Rakotozafy et al. 55 (CAN), **MK780924**. *Tragiatristis* Müll.Arg. Brazil • Anderson 9117 (NY), **MK780981**. *Tragiauberbana* Müll.Arg. Argentina • Zuloaga 5785 (MO), KP794374. *Tragiaurens* L. USA • Abbott 22604 (MO), **MK780957**. *Tragiaurens* L. USA • Anderson 7188 (MO), KP794407. *Tragiaurens* L. USA • Kral 41155 (MO), KP794406. *Tragiaurens* L. USA • Mayfield 2006 (MO), **MK780956**. *Tragiaurens* L. USA • Thomas 100075 (MO), KP794404. *Tragiaurens* L. USA • Thomas 124328 (MO), KP794405. *Tragiaurens* L. USA • Thomas 55051 (CAN), KP794403. *Tragiaurticifolia* Michx. USA • Thomas 97259 (CAN), KP794365. *Tragiavolubilis* L. Bolivia • Peñarando et al. 651 (MO), **MK780987**. *Tragiavolubilis* L. Bolivia • Villalobos et al. 1251 (MO), **MK780988**. *Tragiavolubilis* L. Brazil • Medeiros and Cardinal-McTeague 556 (R), **MK780984**. *Tragiavolubilis* L. Brazil • Medeiros and Cardinal-McTeague 563 (R), **MK780985**. *Tragiavolubilis* L. Brazil • Medeiros et al. 490 (R), **MK780983**. *Tragiavolubilis* L. Brazil • Medeiros et al. 499 (R), **MK780982**. *Tragiavolubilis* L. Costa Rica • Hammel 19108 (MO), KP794362. *Tragiavolubilis* L. Nicaragua • Stevens 29658 (MO), KP794361. *Tragiavolubilis* L. Nicaragua • Stevens and Montiel 34954 (MO), **MK780986**. *Tragiayucatanensis* Millsp. Guatemala • Christenhusz 5678 (MO), KP794384. *Tragiayucatanensis* Millsp. Mexico • Alvarez 4398 (MO), KP794382. *Tragiayucatanensis* Millsp. Mexico • Lira 420 (MO), KP794385. *Tragiellaanomala* (Prain) Pax & K.Hoffm. Tanzania • Luke 11120 (MO), KP794324. *Tragiellaanomala* (Prain) Pax & K.Hoffm. Tanzania • Mwasumbi 16202 (MO), KP794323. *Tragiellafriesiana* (Prain) Pax & K.Hoffm. Zambia • Nkhoma 73 (MO), KP794346. *Tragiellafriesiana* (Prain) Pax & K.Hoffm. Zambia • Nkhoma 74 (MO), KP794347. *Tragiellanatalensis* (Sond.) Pax & K.Hoffm. Tanzania • Massawe 519 (MO), KP794330. *Zuckertiacordata* Baill. Costa Rica • González 1045 (MO), KP794399. *Zuckertiacordata* Baill. Mexico • Calzada 1544 (MO), KP794400. *Zuckertiamanuelii* (V.W.Steinm. & Ram.-Amezcua) Card.-McTeag. & L.J.Gillespie Mexico • Steinmann et al. 6303 (CAN), **MK780989**.

## References

[B1] Baillon H (1858) Étude Générale du Groupe des Euphorbiacées. V. Masson, Paris.

[B2] Berry PE Holst BK Yatskievych K (1995) Introduction. In: Berry PE, Holst BK, Yatskievych K (Eds) Flora of the Venezuelan Guayana. Volume 1: Introduction. Missouri Botanical Garden & Timber Press, Portland, xv–xxii.

[B3] Cardinal-McTeague WM Gillespie LJ (2016) Molecular phylogeny and pollen evolution of Euphorbiaceae tribe Plukenetieae. Systematic Botany 41(2): 329–347. 10.1600/036364416X691759

[B4] Cardinal-McTeague WM Wurdack KJ Sigel EM Gillespie LJ (2019) Seed size evolution and biogeography of *Plukenetia* (Euphorbiaceae), a pantropical genus with traditionally cultivated oilseed species. BMC Evolutionary Biology 19(1): 29. 10.1186/s12862-018-1308-9 30670006 PMC6341577

[B5] Cervantes A Fuentes S Gutiérrez J Magallón S Borsch T (2016) Successive arrivals since the Miocene shaped the diversity of the Caribbean Acalyphoideae (Euphorbiaceae). Journal of Biogeography 43(9): 1773–1785. 10.1111/jbi.12790

[B6] Darriba D Taboada GL Doallo R Posada D (2012) jModelTest 2: More models, new heuristics and parallel computing. Nature Methods 9(8): 772. 10.1038/nmeth.2109 PMC4594756 22847109

[B7] Dorr LJ Romero-Hernández C Wurdack KJ (2018) A new large-flowered species of *Andeimalva* (Malvaceae, Malvoideae) from Peru. PhytoKeys 110: 91–99. 10.3897/phytokeys.110.29376 PMC6232244 30429661

[B8] Gillespie LJ (1994a) Pollen morphology and phylogeny of the tribe Plukenetieae (Euphorbiaceae). Annals of the Missouri Botanical Garden 81(2): 317–348. 10.2307/2992101

[B9] Gillespie LJ (1994b) A new section and two new species of *Tragia* (Euphorbiaceae) from the Venezuelan Guayana and French Guiana. Novon 4(4): 330–338. 10.2307/3391440

[B10] Huft MJ (1989) New and critical taxa of Euphorbiaceae from South America. Annals of the Missouri Botanical Garden 76(4): 1077–1086. 10.2307/2399692

[B11] Katoh K Standley D (2013) MAFFT multiple sequence alignment software version 7: Improvements in performance and usability. Molecular Biology and Evolution 30(4): 772–780. 10.1093/molbev/mst010 23329690 PMC3603318

[B12] Leandri JD (1971) Un sous-genre malgache nouveau de *Tragia* (Euphorbiacées). Adansonia, sér. 2 11: 435–439.

[B13] Medeiros D de Senna Valle L Valka Alves RJ (2013) Revalidation of the genera *Bia* and *Zuckertia* (Euphorbiaceae) with *B.capivarensis* sp. nov. from Serra da Capivara, Brazil. Nordic Journal of Botany 31(5): 595–602. 10.1111/j.1756-1051.2012.01616.x

[B14] Miller MA Pfeiffer W Schwartz T (2010) Creating the CIPRES Science Gateway for inference of large phylogenetic trees. Proceedings of the 2010 Gateway Computing Environments Workshop (GCE), New Orleans, 1–8. 10.1109/GCE.2010.5676129

[B15] Mulgura de Romero M Gutierrez de Sanguinetti M (1989) Actualizacion taxonomica de *Tragia* (Euphorbiaceae) para Argentina y regiones limitrofes. Darwiniana 29(1–4): 77–138.

[B16] Radcliffe-Smith A (2001) Genera Euphorbiacearum. Royal Botanic Gardens, Kew.

[B17] Rambaut A Drummond A Xie D Baele G Suchard M (2018) Posterior summarization in Bayesian phylogenetics using Tracer 1.7. Systematic Biology 67(5): 901–904. 10.1093/sysbio/syy032 29718447 PMC6101584

[B18] Ronquist F Teslenko M van der Mark P Ayres DL Darling A Höhna S Larget B Liu L Suchard MA Huelsenbeck JP (2012) MrBayes 3.2: Efficient Bayesian phylogenetic inference and model choice across a large model space. Systematic Biology 61(3): 539–542. 10.1093/sysbio/sys029 22357727 PMC3329765

[B19] Stamatakis A (2014) RAxML version 8: A tool for phylogenetic analysis and post-analysis of large phylogenies. Bioinformatics (Oxford, England) 30(9): 1312–1313. 10.1093/bioinformatics/btu033 PMC3998144 24451623

[B20] Steinmann VW Ramírez-Amezcua Y (2013) *Biamanuelii* (Euphorbiaceae: Acalyphoideae), a new species from Sierra de Coalcomán, Michoacán, Mexico. Revista Mexicana de Biodiversidad 84(3): 746–750. 10.7550/rmb.32014

[B21] Webster GL (2007) Taxonomic and nomenclatural changes in American Euphorbiacaeae sensu lato. Contributions from the University of Michigan Herbarium 25: 235–239.

[B22] Webster GL (2014) Euphorbiaceae. In: Kubitzki K (Ed.) The Families and Genera of Vascular Plants.Volume XI. Flowering Plants. Eudicots. Malpighiales. Springer Verlag, Berlin and Heidelberg, Germany, 51–216. 10.1007/978-3-642-39417-1_10

[B23] Wurdack KJ Hoffmann P Chase MW (2005) Molecular phylogenetic analysis of uniovulate Euphorbiaceae (Euphorbiaceae sensu stricto) using plastid *rbcL* and *trnL*-*F* DNA sequences. American Journal of Botany 92(8): 1397–1420. 10.3732/ajb.92.8.1397 21646159

